# Anodal transcranial direct current stimulation (tDCS) via the Fp3–Fp2 montage disrupts face recognition skills

**DOI:** 10.1093/oons/kvag010

**Published:** 2026-08-22

**Authors:** Ciro Civile, Guangtong Wang, Amelia Moore

**Affiliations:** Department of Psychology, Faculty of Health & Life Sciences, University of Exeter, Washington Singer Building, Perry Road, Exeter EX4 4QG, United Kingdom; Department of Psychology, Faculty of Health & Life Sciences, University of Exeter, Washington Singer Building, Perry Road, Exeter EX4 4QG, United Kingdom; Department of Psychology, Faculty of Health & Life Sciences, University of Exeter, Washington Singer Building, Perry Road, Exeter EX4 4QG, United Kingdom

**Keywords:** Tdcs, fie, face recognition, perceptual learning, Cfmt

## Abstract

Over the past fifteen years, transcranial direct current stimulation (tDCS) has been used to modulate face recognition skills. One influential line of work has shown that anodal stimulation delivered with the anode at Fp3, a site overlying the left dorsolateral prefrontal cortex (DLPFC), and the return/cathode at Fp2 reduces the face inversion effect (FIE) by selectively disrupting recognition of upright faces, an effect interpreted as the disruption of perceptual learning. A recurring limitation is that the behavioural paradigms employed have relied on stimulus recognition rather than face identity recognition, because the same images were shown at study and test. It therefore remains unclear whether this procedure modulates face identity recognition or merely image-level recognition. Here we addressed this by applying the tDCS procedure during upright and inverted versions of the Cambridge Face Memory Test (CFMT), a robust identity-recognition paradigm in which learned identities must be recognised across novel images varying in viewpoint, lighting, and noise. Participants (N = 64) were randomly assigned in a double-blind, between-subjects design to anodal or sham/control tDCS. A robust FIE was obtained overall, and it was significantly reduced in the anodal group relative to sham. Critically, this reduction was driven by impaired recognition of upright faces under anodal stimulation, with no difference between groups for inverted faces. These results extend the modulation of the FIE from image to face identity recognition, demonstrating that anodal stimulation, via the Fp3–Fp2 montage, disrupts face recognition skills indexed by one of the most widely used tests of face-recognition ability.

## Introduction

Can transcranial direct current stimulation (tDCS) causally change how the human brain remembers and recognises faces? Over the past fifteen years, a number of studies have investigated the effects of tDCS on face recognition, and two main strands of research have emerged that differ chiefly in the electrode montages adopted and the cortical areas nominally targeted. One strand has used predominantly anodal tDCS applied over occipitotemporal and parietal areas that previous work has associated with face-specific brain activation (McKone and Kanwisher 2005), with the aim of enhancing face recognition performance. Differences in tDCS montages, behavioural tasks, and outcome measures across these studies have, however, sometimes led to contrasting results. The other strand has focused on applications of anodal tDCS with the anode placed over the dorsolateral prefrontal cortex (DLPFC) in order to investigate the perceptual learning mechanisms that support face recognition. The specific target site in this second strand was derived from earlier tDCS studies of learning tasks involving prototype-defined artificial stimuli (Ambrus et al. 2011, Kincses et al. 2004) and corresponds to the region identified by neuroimaging as highly activated during the learning of such stimuli (Seger et al. 2000).

In this second strand, the methods and procedures have been highly standardised (double-blind, between-subjects designs with active-control and sham conditions). However, the behavioural tasks used to measure face recognition have focused on stimulus (image) recognition rather than on face identity recognition, which limits the extent to which these findings can be connected to broader advances in the face recognition literature. On this basis, the aim of the present work is to challenge and extend this literature by testing directly whether the anodal tDCS procedure previously developed modulates face recognition when it is measured through a robust identity-recognition paradigm, the Cambridge Face Memory Test (CFMT; Duchaine and Nakayama 2006).

One strand of research has asked whether anodal tDCS over posterior face-responsive cortex can enhance face processing, and several findings suggest that it can. Barbieri et al. (2016) found that offline anodal tDCS over the right occipital cortex (PO8) improved accuracy on tests of face and object perception and memory (including the CFMT and the Cambridge Car Memory Test) relative to sham; the enhancement was site-specific (stimulation over sensorimotor cortex had no effect) but neither category-specific nor process-specific, affecting faces and objects, perception and memory alike. Brunyé et al. (2017) reported that anodal tDCS targeting the right fusiform gyrus (PO10) selectively increased working-memory capacity for faces but not houses, with the largest gains at the highest memory loads and in participants with lower baseline capacity. More recently, Saghravanian and Esteky (2025) found that bilateral occipitotemporal stimulation (anode over the right, cathode over the left hemisphere) improved identification of ambiguous morphed faces, an effect they interpreted as interhemispheric rebalancing favouring the right-hemisphere dominance in face processing.

These enhancement effects are not uniform, however: whether they emerge, and in which direction, depends on the task, the montage, and participants’ baseline ability. Wu et al. (2021) found no group-level effect of high-definition tDCS over the left or right fusiform face area (FFA) or the left superior temporal sulcus on face-view discrimination; instead, baseline performance predicted the direction of change under anodal stimulation of the left FFA, with better performers worsening and poorer performers improving, a ‘convergence effect’ whose opposing individual changes cancel out at the group level. Kho et al. (2023) found that multifocal stimulation of the FFA enhanced recognition of facial features but not of whole faces, whereas stimulation of the occipital face area (OFA) had no effect on either. Renzi et al. (2015) even reported that anodal tDCS over the right OFA interfered with the detection of degraded (Mooney) faces and objects, while leaving holistic processing in the composite-face task unaffected. Taken together, this first strand indicates that anodal tDCS over occipitotemporal and adjacent areas can enhance face processing, but that such effects are moderated by task demands, stimulation parameters, and individual differences in baseline ability (see also Willis et al. 2019).

Concurrently, a second series of studies used double-blind, between-subjects designs to examine the effect of anodal tDCS delivered with the anode at Fp3 (a site overlying the left DLPFC) on perceptual learning as indexed by the inversion effect. The inversion effect for faces (Diamond and Carey 1986, Yin 1969) refers to reduced recognition performance for faces presented upside down relative to faces in their usual upright orientation. An analogous inversion effect can be obtained for non-mono-oriented artificial stimuli, prototype-defined checkerboards, that participants have never encountered before entering the laboratory and for which prior expertise can therefore be controlled. This checkerboard inversion effect has been used as an index of perceptual learning (Civile et al. 2014, McLaren and Civile 2011). Using this approach, these studies demonstrated that anodal stimulation at Fp3 can reduce the inversion effect specifically by impairing performance for upright stimuli. Anodal tDCS delivered at Fp3 (return/cathode electrode at Fp2) reduces the inversion effect for faces as well as the inversion effect for checkerboards, relative to sham, in both cases by disrupting performance for upright stimuli, with no differences between anodal and sham in the inverted conditions (Civile, McCourt, and McLaren 2024, Civile et al. 2021, Civile et al. 2021, Civile et al. 2020, Civile et al. 2018, Civile et al. 2016).

Importantly, these key findings were replicated and the investigation extended to active-control conditions. For instance, Civile et al. (2018) showed that anodal tDCS at Fp3 (return/cathode at Fp2) reduced the FIE relative to sham, due to reduced performance for upright faces, whereas an active-control condition in which the anode targeted the right inferior frontal junction (rIFJ; return/cathode at Fp1) left the FIE as robust as in the sham group. Civile et al. (2021) added a further active-control condition in which anodal tDCS targeted the occipito-parietal area (PO8) while the return/cathode remained at Fp2, exactly as in the established Fp3–Fp2 montage. This control condition did not differ from sham, whereas the Fp3–Fp2 montage replicated the results of Civile et al. (2018). Finally, Civile, McCourt, and McLaren (2024) directly compared the effects of the anodal Fp3–Fp2 montage with those of an anodal Fp3–Cz montage on the inversion effects for faces and checkerboards. Only the Fp3–Fp2 montage reduced the two inversion effects relative to sham; the Fp3–Cz montage behaved like sham. Taken together, these studies show that placing both electrodes over different areas does not modulate the inversion effect (Civile et al. 2018), that moving the anode away from Fp3 while keeping the return at Fp2 has no effect (Civile et al. 2021), and that keeping the anode at Fp3 while moving the return from Fp2 to Cz also has no effect (Civile, McCourt, and McLaren 2024). Thus, modulation of the inversion effect is obtained only when the anode targets Fp3 and the return/cathode is placed over the contralateral supraorbital area (Fp2).

The parallel effects of the same tDCS procedure on the inversion effects for faces and checkerboards, motivated an explanation based on perceptual expertise expressed through perceptual learning.

The anodal tDCS procedure was subsequently extended to investigate the perceptual expertise mechanisms underlying biases in face recognition, such as the own-race bias (Meissner and Brigham 2001) and the own-age bias (Hills and Lewis 2011). These studies showed that disrupting expertise for the own-race or own-age upright-face condition through anodal tDCS at Fp3 (return at Fp2) reduced the inversion effect for own faces and, as a consequence, eliminated the interaction (inversion × race, or inversion × age) that indexes these robust phenomena (Civile and McLaren 2022, Civile and Wang 2026).

Finally, Civile et al. (2020) and Civile, Waguri, and McLaren (2024) further demonstrated the efficacy of the Fp3–Fp2 tDCS procedure by combining tDCS and electroencephalography (EEG) simultaneously. This work revealed that the behavioural changes in the FIE are accompanied by changes in event-related potentials (ERPs), specifically the N170, a component robustly associated with the mechanisms underlying the FIE (Eimer 2011), as well as by analogous inversion effects for prototype-based stimuli with which participants had been familiarised (Rossion et al. 2002).

The theoretical framework guiding this research programme is the McLaren, Kaye and Mackintosh (MKM) model of perceptual learning (McLaren et al. 1989, McLaren and Mackintosh 2000, McLaren et al. 2012). On this account, experience with a prototype-defined category, such as upright faces, reduces the salience of the features common to its exemplars while leaving each exemplar’s distinctive features relatively salient, thereby improving discrimination between exemplars. The inversion effect arises because this salience-modulation benefit accrues only for the familiar upright orientation, and anodal tDCS at Fp3 is held to disrupt the salience-modulation mechanism itself, selectively impairing upright performance. This framework generates the prediction tested here: if the tDCS procedure acts on the expertise-based learning that supports the individuation of faces, its behavioural signature — disruption of upright but not inverted performance — should extend from image recognition to identity recognition as measured by the CFMT.

One limitation raised regarding this body of work is whether it actually measures face recognition rather than image/stimulus recognition. Because the behavioural tasks used never presented different images of the same face identity, the procedure has not yet been tested with a robust face identity-recognition task of the kind used to assess prosopagnosia, such as the CFMT (Duchaine and Nakayama 2006). In the present study we address this issue by applying the same anodal tDCS procedure during the CFMT. Confirming the previous pattern of results would demonstrate that anodal tDCS delivered via the Fp3–Fp2 montage indeed disrupts face identity-recognition performance. By contrast, a failure to obtain this extension would provide a first clear indication that this tDCS procedure specifically affects stimulus (image) recognition rather than face identity recognition.

## Materials and methods

### Participants

Sixty-four naïve participants (46 women; mean age = 22.8 years; range = 19–35 years) took part in the study. Participants were recruited through the SONA system, which includes members of the community and university students, and were screened in accordance with established safety criteria for tDCS. Informed consent was obtained from all participants. The study was conducted in accordance with the principles of the Declaration of Helsinki and was approved by the Psychology Research Ethics Committee at the University of Exeter.

The sample size was determined via an a priori power analysis conducted using G*Power (Faul et al. 2007), targeting a medium effect size for the critical Face Orientation (upright, inverted) × tDCS (anodal, sham) interaction with power of at least .80. In addition, the design took into account the recommendations of Brysbaert and Stevens (2018) regarding statistical power in repeated-measures designs. Drawing on sampling simulations from two mega-studies, they concluded that approximately 1600 observations (across participants and trials) in the smallest analysis cell are sufficient to detect smaller-than-medium effects with power exceeding .80. In the present study, the smallest analysis cell comprised 72 observations per participant across 64 participants, yielding 4608 observations.

### Materials

The stimuli consisted of faces of young male Western Caucasians with neutral expressions selected from the Chicago Face Database and the FACES database (Ebner et al. 2010, Ma et al. 2015). Each original 2D image was transformed into a 3D model using FaceGen Artist software, after which lighting and pose were manipulated in Daz Studio; this process automatically removed hair and facial blemishes. The resulting images were then converted to greyscale, cropped, and overlaid with Gaussian noise using a custom Python script. Each face identity was processed to generate 12 distinct variations differing in viewing angle and lighting, consistent with previous versions of the task (Duchaine and Nakayama 2006, McKone et al. 2011, 2012). The 12 conditions were: front view; left 30°; right 30°; left 60°; right 60°; front view–up light; front view–left light; right 30°–right light; front view–down light with noise; front view–right light with noise; left 30°–right light with noise; and right 60°–left light with noise.

Upright and inverted versions of the CFMT were created to measure the FIE, using two different stimulus sets with distinct face identities. As in the original CFMT (Duchaine and Nakayama 2006), each stimulus set comprised 6 target identities and 48 distractor identities, each presented in the 12 conditions (hereinafter ‘conditions 1–12’). Each set includes two more distractor identities than the original version. This modification was necessary because the overall design requires 144 distractors (72 trials × 2 distractors per trial): using 48 distractor identities allows each identity to appear exactly three times (48 distractor identities × 3 appearances).

### tDCS apparatus

Stimulation was delivered using a battery-powered, constant-current stimulator (Neuroelectrics; https://www.neuroelectrics.com) via a pair of saline-soaked surface sponge electrodes (35 cm^2^) placed on the scalp at the designated sites. A bilateral, bipolar, non-balanced montage was used, with the anodal electrode positioned over the target site (Fp3) and the return electrode over the contralateral supraorbital region above the right eyebrow (Fp2), consistent with previous studies using the same protocol (Civile and McLaren 2022, Civile et al. 2021, Civile and Wang 2026). The procedure was implemented using the system’s built-in double-blind functionality.

In the active (anodal) condition, a direct current of 1.5 mA was applied for 10 minutes, including a 5-second fade-in and a 5-second fade-out. Stimulation commenced at the onset of the first task and continued for 10 minutes. Across the two experimental tasks (upright and inverted faces), the total duration of the study was approximately 35 minutes. The effects of tDCS are known to persist for up to 120 minutes following stimulation (Jamil et al. 2017, Nitsche and Paulus 2000). In the sham condition, participants experienced identical 5-second fade-in and fade-out periods, but only 30 seconds of stimulation were delivered during the intervening interval.

### Procedure: The Cambridge face memory test (CFMT)

We adapted our procedure to one of the most established paradigms used to measure face-recognition ability, the CFMT originally developed by Duchaine and Nakayama (2006). The experiment consisted of two tasks (upright and inverted versions), each comprising four phases: practice, introduction/same images (18 trials), novel images (30 trials), and novel images with noise (24 trials). The two tasks took approximately 35 minutes to complete, and their order of presentation was counterbalanced across participants.

In each task, to ensure balanced presentation, the 48 distractor identities were organised into fixed pairs (e.g. distractor identity 1 always appeared with distractor identity 2). These distractor pairs were systematically rotated across target identities and lighting/angle conditions. Specifically, the assignment was counterbalanced such that a given distractor pair (e.g. distractors 1 and 2) was not exclusive to a single target identity; instead, each pair appeared with multiple target identities across varying conditions—for example, with target identity 1 in condition 1 (front view), target identity 3 in condition 9 (front view–down light with noise), and target identity 6 in condition 5 (right 60°). This design ensured that distractor identity was orthogonal to both target identity and the specific visual condition. The relative location of the target was counterbalanced across the 72 test trials such that the target appeared on the left, in the middle, and on the right in 24 trials each.

#### Practice

The practice phase familiarised participants with the procedure. Participants were introduced to three facial images of a cartoon character. The left 30°, front-view, and right 30° images were presented sequentially, each for 3 s and preceded by a 0.5-s blank screen. Three test trials followed. In the first, the left 30° image was displayed together with left 30° images of two distractor identities on the same screen for 4 s (preceded by a 0.5-s blank screen), and participants identified the face they had just seen by pressing keys 1, 2, or 3. Two additional test trials followed for the front-view and right 30° images.

#### Introduction/same images

This phase involved the six target identities. As in the practice phase, each target identity included three learning trials and three test trials. Trials for each target identity were grouped into a block, resulting in six blocks presented in random order.

#### Novel images

This phase began with a learning trial in which a composite review image containing all six target identities was presented for 20 s (preceded by a 0.5-s blank screen). Thirty test trials were then presented in random order. In each trial, one target identity was displayed together with two distractor identities on the same screen for 4 s (preceded by a 0.5-s blank screen), as in the previous test phases. On each screen the two distractor identities always matched the condition of the target. Participants selected the face belonging to one of the six identities they had learned. In contrast to the same-images phase, the target identities were now presented in five new conditions involving variations in lighting, pose, or both: left 60°, right 60°, front view–up light, front view–left light, and right 30°–right light. Each distractor identity appeared in the same five conditions as the targets.

#### Novel images with noise

This phase began with the same learning trial as the novel-images phase. Twenty-four test trials were then presented in random order, following the same procedure. Here, the six target identities appeared in four conditions containing Gaussian visual noise: front view–down light with noise, front view–right light with noise, left 30°–right light with noise, and right 60°–left light with noise. Each distractor identity appeared in the same four conditions as the targets. Within each test trial, the level of noise was identical for the target and the two distractors. Noise was added for two reasons. First, by the beginning of this final phase participants had already seen each target 13 times, so the noise served to keep performance away from ceiling. Second, studies with non-prosopagnosic participants indicate that noise increases reliance on configural processing, a mechanism on which face recognition normally depends (Duchaine and Nakayama 2006; Maurer et al. 2002; McKone et al. 2001).

## Results

The primary measure was the cumulative accuracy score (Duchaine and Nakayama 2006) across the three test phases (same images, novel images, and novel images with noise) for each task (upright and inverted) in the anodal and sham groups.

For the main analysis we conducted a 2 × 2 mixed-model ANOVA with Face Orientation (upright, inverted) as a within-subjects factor and tDCS (sham, anodal) as a between-subjects factor. There was a main effect of Face Orientation, *F*(1, 62) = 69.68, *p* < .001, η^2^_p_ = .53, indicating a robust inversion effect overall, with upright faces (*M* = 41.2, *SD* = 7.5) recognised better than inverted faces (*M* = 32.5, *SD* = 5.6). Critically, the Face Orientation × tDCS interaction was significant, *F*(1, 62) = 7.04, *p* = .010, η^2^_p_ = .10: the inversion effect in the anodal group (*M* = 5.9, *SD* = 8.4) was significantly reduced relative to the inversion effect in the sham group (*M* = 11.4, *SD* = 8.1). A significant main effect of tDCS was also found, *F*(1, 62) = 5.52, *p* = .022, η^2^_p_ = .08, indicating that overall performance was lower in the anodal group (*M* = 35.4, *SD* = 5.2) than in the sham group (*M* = 38.3, *SD* = 4.5).

**Figure 1:**
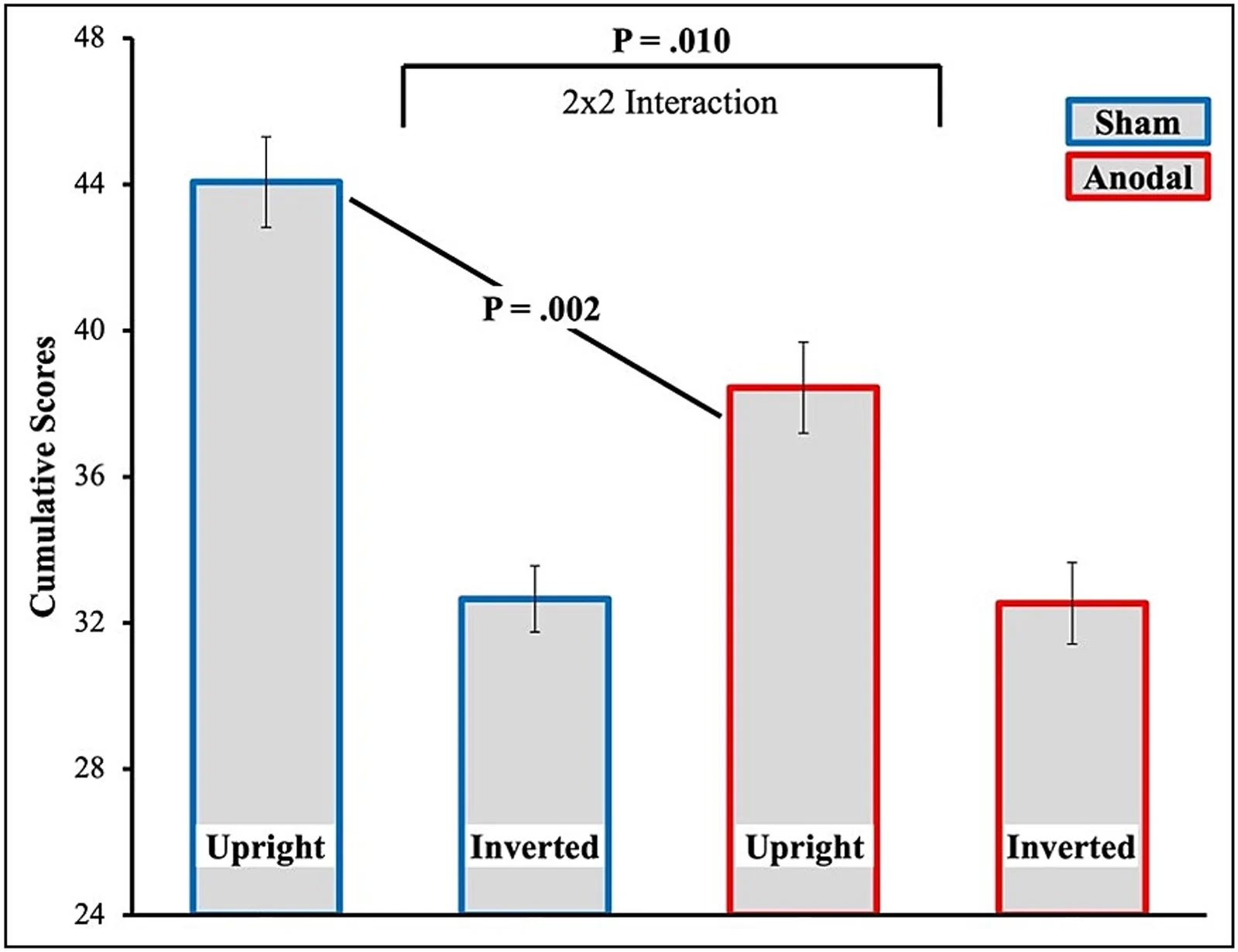
The x-axis represents the face stimuli in each tDCS condition. The y-axis represents the mean cumulative scores. Error bars are SEM.

In line with all previous work applying the same tDCS procedure to the inversion effect (e.g. Civile et al. 2018, Civile, Quaglia, et al. 2021), we conducted two independent-samples *t*-tests. Performance for upright faces was significantly disrupted in the anodal group (*M* = 38.4, *SD* = 7.1) relative to the sham group (*M* = 44.1, *SD* = 6.9), *t*(62) = 3.20, *p* = .002, *d* = 0.80. By contrast, performance for inverted faces did not differ between the anodal group (*M* = 32.5, *SD* = 6.3) and the sham group (*M* = 32.6, *SD* = 5.1), *t*(62) = 0.08, *p* = .93, *d* = 0.02 (Fig. 1).

Additional analyses examining performance across the three test phases (same images, novel images, and novel images with noise) separately for upright and inverted faces, and examining the inversion effect across phases, are reported in the Supplementary Material. These analyses confirm that the disruption of upright-face recognition by anodal tDCS was comparable in magnitude across phases (no Phase × tDCS interaction), and that no effect of tDCS was present for inverted faces at any phase.

## Discussion

The present study set out to determine whether the anodal tDCS procedure that reliably reduces the FIE in stimulus-recognition tasks also modulates face recognition when performance is measured with a robust identity-recognition paradigm. Using upright and inverted versions of the CFMT, we obtained a clear answer. Anodal tDCS delivered via the Fp3–Fp2 montage significantly reduced the inversion effect relative to sham, and this reduction was driven entirely by impaired recognition of upright faces, with no difference between groups for inverted faces. This is the same signature, selective disruption of upright performance leaving inverted performance intact, that has been reported across earlier applications of this procedure (e.g. Civile et al. 2018; Civile et al. 2021). The novelty here is that the effect now emerges on a task in which learned identities must be recognised across novel images that vary in viewpoint, lighting, and added noise, so that success cannot be achieved through image matching alone.

For the tDCS literature, these findings make two contributions. First, they close an important gap of construct validity. A standing question about this tDCS procedure was whether it modulated genuine face identity recognition or only image-level recognition, given that previous paradigms presented the same images at study and test. Because the CFMT requires generalisation across novel images of each identity, the present disruption of upright-face performance cannot be reduced to impaired image recognition; it reflects a change in the recognition of face identity. The Fp3–Fp2 procedure therefore joins the small set of tDCS manipulations that causally influence face identity processing, alongside reports of enhancement following occipitotemporal or fusiform stimulation (Barbieri et al. 2016, Brunyé et al. 2017, Saghravanian and Esteky 2025). Second, the results sharpen an apparent divergence within the tDCS-and-faces literature. Studies targeting posterior face-selective cortex have generally reported enhancement of face and object processing (Barbieri et al. 2016, Brunyé et al. 2017), although such enhancements are not always obtained and can depend on baseline performance, task difficulty, and montage (Kho et al. 2023, Willis et al. 2019, Wu et al. 2021). By contrast, anodal stimulation via the Fp3–Fp2 montage consistently produces disruption of upright-face recognition. Rather than being contradictory, these outcomes are consistent with the two strands acting on different computations: posterior stimulation modulates the perceptual encoding of faces within face-selective cortex, whereas Fp3–Fp2 stimulation modulates the learning-based process that assigns salience to diagnostic features. The convergence and baseline-dependence effects reported for posterior sites (Wu et al. 2021) further indicate that group-level null results at those sites need not imply the absence of a causal role, reinforcing the value of the Fp3–Fp2 procedure, which yields a reliable group-level effect that is reversible by cathodal stimulation (Civile et al. 2025).

For the FIE literature, the present results speak to the long-running debate over whether the inversion effect reflects a face-specific mechanism or domain-general perceptual expertise (Diamond and Carey 1986, Yin 1969). The fact that a single stimulation protocol reduces the inversion effect for faces and for prototype-defined checkerboards alike (Civile et al. 2016), and does so by the same means, impairing upright but not inverted performance, supports an expertise-based account in which the inversion effect arises from experience-driven perceptual learning that is available for upright but not for inverted stimuli. The demonstration that this modulation extends to identity recognition on the CFMT strengthens that account, because the CFMT indexes precisely the kind of individuating expertise that inversion is thought to disrupt. The present findings also complement the electrophysiological evidence that this procedure alters the N170 (Civile, Waguri, and McLaren 2024, Civile, Waguri, et al. 2020), a component whose sensitivity to inversion is one of the most reliable neural markers of the inversion effect (Eimer 2011, Rossion et al. 2002). Together, the behavioural and neural evidence converge on the view that the inversion effect can be experimentally down-regulated at its perceptual learning source, and that this is now observable on a standard test of individual face recognition.

More broadly, the results contribute to the face recognition literature in two ways. First, they connect the Fp3–Fp2 tDCS research programme to the mainstream psychometrics of face recognition. The CFMT is among the most widely used measures of individual differences in face-recognition ability and is central to the assessment of developmental prosopagnosia and of own-group biases (Duchaine and Nakayama 2006, McKone et al. 2011, 2012). Showing that the procedure moves performance on this instrument provides a common currency in which the perceptual-learning account can be compared with other neurocognitive models of face recognition. Second, the finding that expertise for upright faces can be temporarily and reversibly reduced offers a causal tool for studying the experience-based components of face recognition, including the own-race and own-age biases that have already been linked to this procedure (Civile and McLaren 2022, Civile and Wang 2026, Hills and Lewis 2011, Meissner and Brigham 2001). Because these biases are widely understood as consequences of differential perceptual experience, a manipulation that selectively targets experience-based expertise provides leverage that correlational designs cannot.

Our results are consistent with, and can be explained by, the MKM model of perceptual learning (McLaren and Mackintosh 2000, McLaren et al. 1989, McLaren et al. 2012). This model attributes perceptual learning and, by extension, the inversion effect, to the modulation of stimulus-element salience by prediction error. It is parameterised such that features which are well predicted by other features (low error) come to have lower salience (activation), whereas features that are not well predicted, such as those unique to an individual exemplar, retain higher salience. This differential salience facilitates discrimination between exemplars that share common features once those exemplars have been pre-exposed, because the shared (common) features are better predicted, and hence less salient, than the distinctive (unique) features.

Extending this to prototype-defined categories, the prototype constitutes the set of common features, while the distinctive features of each exemplar are unique and differ across exemplars. Familiarity with such a category reduces generalisation between exemplars because the influence of the common features declines with exposure, lowering their salience. Discrimination therefore improves, because the unique features remain relatively salient. This salience modulation operates only for upright stimuli, because participants have far less experience with inverted stimuli and so do not benefit from comparable perceptual learning.

Applied to faces, our extensive experience with upright faces means that common facial features become strongly associated with one another, reducing their prediction error and, in turn, the salience of the units representing them. The novel, unpredicted features specific to a given face do not suffer this reduction; they stand out and become available for learning, improving discrimination among upright faces. When faces are inverted, this mechanism no longer applies, because the unfamiliar spatial arrangement renders features less well predicted by one another, disrupting the salience modulation that normally distinguishes common from unique features of upright faces.

Under this account, anodal tDCS reduces the inversion effect by impairing performance for upright faces: the stimulation disables the salience-modulation mechanism, so that prior exposure to a prototype-defined category, instead of enhancing the discriminability of its exemplars, now enhances generalisation between them. Features common to exemplars become more prominent because they co-activate one another, while unique features remain low in salience because they receive no additional activation. It is this change in perceptual learning that reduces participants’ ability to discriminate between upright faces, effectively making the faces appear more ‘similar’, and thereby reduces the inversion effect. This explanation is supported by the simulation work in Civile et al. (2023) and by experimental work using the same tDCS procedure in a prototype-distortion learning task, in which increased salience of prototypical features within familiar stimuli led to improved task performance (McLaren et al. 2016).

One might argue that our results could instead be explained by anodal tDCS altering the processing that occurs during the encoding of faces, rather than the mechanisms of perceptual learning. Civile et al. (2025) tested this directly using an old/new recognition task in which participants received anodal stimulation during the encoding (study) phase followed by either sham, the reverse polarity (cathodal), or a matched control at recognition. The inversion effect was significantly reduced, through disrupted recognition of upright faces, in the group that received anodal tDCS followed by sham at recognition, whereas the inversion effect in the anodal-then-cathodal group returned to the robust level found in the sham–sham group. These modulatory effects, supported by accompanying simulation work, suggest that cathodal stimulation removes the increased generalisation induced by anodal tDCS by rebalancing the feature-salience modulation mechanism. During anodal tDCS, the salience of common features is kept relatively high, making it easier for the observer to focus on the similarities between faces (thereby disrupting recognition). When cathodal tDCS is then applied, it counteracts the effect of anodal tDCS on the salience of common features (which returns to low), allowing observers to focus on the unique features that are diagnostic of each individual face. Crucially, if anodal tDCS affected only encoding, applying cathodal tDCS during the recognition task would be too late to reverse performance, yet it does reverse it, indicating that the locus of the effect is the perceptual-learning process rather than encoding per se.

A further issue concerns what the present findings can and cannot establish about the distinction between image-level and identity-level processing (Bruce 1982, Bruce and Young 1986). We acknowledge that showing that tDCS disrupts performance on a task requiring recognition across different images does not, by itself, demonstrate that identity-recognition mechanisms are affected: identity recognition necessarily depends on earlier image-level processes, including visual analysis, perceptual discrimination, and attention to diagnostic facial information, and a manipulation disrupting any of these shared upstream processes would also impair performance on an identity-recognition task. However, the selectivity of the effect speaks against a purely upstream account. A manipulation acting on general visual or attentional processes should impair recognition of inverted faces as well as upright ones, whereas anodal tDCS impaired only upright-face recognition. Because inversion disrupts configural processing (Maurer et al. 2002, McCourt et al. 2023), this selectivity indicates that the stimulation affects, at least in part, a mechanism involved in face recognition — specifically configural processing — which is precisely the logic underlying the use of the inversion manipulation in the original validation of the CFMT (Duchaine and Nakayama 2006).

The novel-images-with-noise phase of the CFMT speaks directly to this point. This phase was designed to test reliance on configural processing, because the noise manipulation disrupts featural processing while leaving configural processing relatively unaltered (Duchaine and Nakayama 2006, McKone et al. 2001). An additional analysis restricted to this phase (see Supplementary Material) showed that anodal tDCS significantly reduced the FIE relative to sham, and that this reduction was driven by impaired performance for upright faces, with no group difference for inverted faces (for which configural processing is in any case disrupted by the rotation manipulation). Thus, even under stimulus conditions designed to isolate configural processing, anodal tDCS disrupted upright-face recognition.

This does not imply that the mechanisms affected are specific to faces: on the perceptual-learning account developed above, compara4ble effects should obtain for any prototype-defined category sharing a common configuration, and the present results do not directly adjudicate the expertise versus face-specificity debate. What they do show is that a tDCS procedure known to modulate the inversion effect for faces and checkerboards alike extends to a paradigm testing identity recognition rather than stimulus recognition. Future work should apply the same procedure to CFMT-format tests adapted to non-face objects, such as the Cambridge Car Memory Test (Dennett et al. 2012), to address the specificity question directly. Future studies could also probe the earlier stages of processing by adopting the combined tDCS–EEG methodology (Civile et al. 2020, Civile, Waguri, and McLaren 2024), examining the effects of stimulation on the inversion effects indexed by the P1, N170, and P250 components. For now, the present study is, to our knowledge, the first to demonstrate that a specific tDCS montage can disrupt face-recognition performance across tasks measuring stimulus recognition and tasks measuring face-identity recognition.

A final note concerns the anatomical specificity of the tDCS procedure and the question of which cortical areas are affected by the Fp3–Fp2 montage. Computational modelling of the electric fields induced by tDCS has repeatedly shown that the current delivered through conventional bipolar montages is spatially diffuse, and that current density is often not maximal at the nominal cortical target, being shaped by anatomical factors such as skull and cerebrospinal-fluid thickness, gyral depth, and inter-electrode distance (Datta et al. 2009). These factors also vary substantially across individuals, producing considerable inter-subject variability in the electric fields induced by the same montage (Laakso et al. 2015). Future work should apply current-flow modelling to this specific montage and, ideally, individualise stimulation parameters based on participants’ anatomy; combined tDCS–fMRI protocols could further help identify the cortical regions mediating the effects reported here.

## Conclusion

Applying an established anodal tDCS procedure (Fp3-Fp2) during upright and inverted versions of the CFMT, we found that stimulation reduced the FIE by selectively disrupting recognition of upright faces. This extends the tDCS-induced modulation of the inversion effect from image recognition to genuine face identity recognition, demonstrates that the tDCS procedure disrupts face recognition skills on one of the field’s standard instruments, and reinforces an account of the inversion effect grounded in experience-based perceptual learning. In doing so, the study connects the tDCS, face inversion, and broader face recognition literatures, and provides a causal, reversible tool for probing the experience-dependent components of human face recognition.

## Ethics

This research was approved by the Psychology Research Ethics Committee at the University of Exeter and was conducted in accordance with the principles of the Declaration of Helsinki. Written informed consent was obtained from all participants.

## Supplementary Material

Supplementary_material_kvag010

## Data Availability

The data underlying this article will be shared on reasonable request to the corresponding author, and are available in an open repository (https://osf.io/y4j5k).
