# Supplementary material for: Anodal transcranial direct current stimulation (tDCS) via the Fp3–Fp2 montage disrupts face recognition skills
Source: Oxf Open Neurosci. 2026 Aug 22;5:kvag010. doi: 10.1093/oons/kvag010 (PMC13537463; doi:10.1093/oons/kvag010)
Supplement: Supplementary file 1 [file supplementary_material_kvag010.zip › Supplementary_file_for_export 1_kvag010.docx]

06-Aug-2026

Dear Dr. Civile,

Thank you for submitting Manuscript ID OXFNSC-2026-038 entitled "Anodal Transcranial Direct Current Stimulation (tDCS) at the DLPFC Disrupts Face Recognition Skills tDCS and face recognition" to Oxford Open Neuroscience.

Your manuscript has been evaluated by two reviewers with relevant expertise. Their comments are included at the bottom of this letter.

The reviewers have raised some substantial concerns that preclude acceptance of your manuscript in its current form. However, I believe that this work could potentially be accepted if revised appropriately. Therefore, I invite you to respond to the reviewer(s)' comments and revise your manuscript within the next 60 days from today date.

There are three key issues raised by the reviewers that I would like you to consider (potentially by clearly including caveats to acknowledge these points):

- Reviewer 1 highlights that data presented in this manuscript does not establish domain specificity for faces

- Reviewer 1 also makes the point that these data cannot establish specificity for anodal excitation of DLPFC, rather than cathodal inhibition at fronotpolar cortex

- Reviewer 2 highlights that your data do not establish that stimulation specifically disrupts identity recognition rather than the image-level processes

Please note that this journal operates with transparent peer review. This means that if your submission is accepted for publication, the full peer review history of your article will publish online alongside your article. This includes reviewer comments, editor decision letters, and your author responses.

To revise your manuscript, log into https://mc.manuscriptcentral.com/oxfnsc and enter your Author Centre, where you will find your manuscript title listed under "Manuscripts with Decisions."  Under "Actions," click on "Create a Revision."  Your manuscript number has been appended to denote a revision.

You may also click the below link to start the revision process (or continue the process if you have already started your revision) for your manuscript. If you use the below link you will not be required to login to ScholarOne Manuscripts.

*** PLEASE NOTE: This is a two-step process. After clicking on the link, you will be directed to a webpage to confirm. ***

https://mc.manuscriptcentral.com/oxfnsc?URL_MASK=d2cbbd6f5d6c4ff288a9f3830a553660

You will be unable to make your revisions on the originally submitted version of the manuscript.  Instead, revise your manuscript using a word processing program and save it on your computer.  Please also highlight the changes to your manuscript within the document by using the track changes mode in MS Word or by using bold or colored text.

<strong>REMINDER:</strong> if you have not already done so, when submitting your revised manuscript, please include a short alternative-text description under the figure legend for each figure. Alt-text is required to ensure the accessibility of our publications. Guidance is available on the journal’s Author Guidelines webpage and at <a href="https://academic.oup.com/pages/for-authors/journals/preparing-and-submitting-your-manuscript" target="_new">https://academic.oup.com/pages/for-authors/journals/preparing-and-submitting-your-manuscript</a>

Once the revised manuscript is prepared, you can upload it and submit it through your Author Centre.

When submitting your revised manuscript, you will be able to respond to the comments made by the reviewer(s) in the space provided.  You can use this space to document any changes you make to the original manuscript.  In order to expedite the processing of the revised manuscript, please be as specific as possible in your response to the reviewer(s).

IMPORTANT:  Your original files are available to you when you upload your revised manuscript.  Please delete any redundant files before completing the submission.

Because we are trying to facilitate timely publication of manuscripts submitted to the Oxford Open Neuroscience, your revised manuscript should be uploaded within the next 60 days from today date.  If it is not possible for you to submit your revision in a reasonable amount of time, we may have to consider your paper as a new submission.

Once again, thank you for submitting your manuscript to the Oxford Open Neuroscience and I look forward to receiving your revision.

Sincerely,
Professor Sam Gilbert
Senior Editor, Oxford Open Neuroscience
sam.gilbert@ucl.ac.uk


Reviewer: 1

Comments to the Author
Here, the authors investigate how tDCS applied to bilateral prefrontal regions modulates performance on the Cambridge Face Memory Test (CFMT). The authors report a significant Face Orientation × tDCS interaction, driven by impaired recognition of upright faces under active stimulation with no corresponding effect on inverted faces. They interpret these findings as causal evidence for the left DLPFC’s involvement in normative face recognition.  The use of tDCS to investigate cognitive and perceptual domains is a worthwhile pursuit, but this manuscript suffers from critical limitations in theoretical grounding, literature integration, and experimental design. Several substantive concerns with the manuscript are detailed below:  

1. Lack of literature integration and theoretical synthesis in the Introduction:
The Introduction currently reads as a sequential list of prior studies and their study-specific results, rather than a cohesive, hypothesis-driven narrative. The Introduction should be restructured to condense the current lengthy blocks of text summarizing individual studies into a series of integrated, thematically organized paragraphs—which would be much better suited to establishing the theoretical implications and hypothesis justification for the current study.  
For example, the McLaren perceptual learning model is introduced late in the Discussion, but is not used to help set up the study in the Introduction. Bringing this theoretical framework forward to earlier in the paper would greatly help situate the motivation and potential theoretical significance of this experiment.  

2. Specificity of the behavioral results:
The DLPFC (and the contralateral frontopolar cathode site) are critical domain-general cognitive hubs for numerous processes engaged by this task, including working memory maintenance, attentional allocation, and rule-based decision-making. While inverted faces are included as a control orientation, baseline recognition for inverted faces is substantially lower (M = 32.5 vs. M = 41.2), meaning this condition does not match the configural working memory load of upright faces. Without a true "positive control" task using a non-face visual category of comparable cognitive difficulty, it is impossible to determine whether these effects are genuinely domain-specific to face recognition per se. This ambiguity significantly limits the scientific impact of the study from a psychological standpoint.  

3. PFC functional anatomy and polarity confounds:
The study utilizes a bilateral, bipolar tDCS montage with the anode placed over the left DLPFC (Fp3) and the cathode over the contralateral frontopolar/supraorbital cortex (Fp2). Without a flipped montage (anode at Fp2, cathode at Fp3), an extracephalic return electrode, or another "active control" tDCS condition, it is unclear whether the observed behavioral disruptions are being driven by anodal excitation at the DLPFC, cathodal inhibition at the frontopolar cortex, or both. This confound greatly restricts the neuroscientific validity and impact of these findings.

Reviewer: 2

Comments to the Author
Dear Editor,
Thank you very much for the opportunity to review this manuscript.
In “Anodal Transcranial Direct Current Stimulation (tDCS) at the DLPFC Disrupts Face Recognition Skills,” the authors investigate whether anodal tDCS delivered at Fp3, intended to target the left dorsolateral prefrontal cortex, disrupts face-identity recognition. The study builds on previous research showing that stimulation using this montage impairs recognition of upright faces in paradigms in which the same facial image is presented during the study and test phases, so they rely more on image recognition than on face recognition. The authors argue that using the Cambridge Face Memory Test, in which identities must be recognised across different images, allows them to establish that the effect extends beyond image recognition to genuine face-identity recognition. This distinction is the principal motivation for the study.
The distinction between image recognition and face-identity recognition is a longstanding one in the face-processing literature (e.g., Bruce, 1982; Bruce & Young, 1986). It is generally accepted that, to investigate recognition of facial identity rather than memory for a particular photograph, different images of the same identity should be presented at learning and test. From this perspective, the authors’ rationale initially appears reasonable: if the aim is to investigate identity recognition, performance should be tested using novel images of the learned identities.
However, I believe that the manuscript overlooks an important logical problem. Presenting different images is a necessary methodological feature of an identity-recognition task, but it is not sufficient to establish that an experimental manipulation acts specifically upon identity-level representations or identity-recognition mechanisms. Face-identity recognition necessarily depends on earlier image-level processes, including visual analysis, perceptual discrimination, attention to diagnostic facial information, and the encoding of the presented image. If Fp3 stimulation disrupts any of these shared upstream processes, as previous research cited by the authors has widely supported, performance will also be impaired on a task requiring recognition across different images. Such a finding would demonstrate that the effect generalises to performance on an identity-recognition task, but not that stimulation specifically disrupts identity recognition rather than the image-level processes upon which identity recognition depends.
An analogy would be demonstrating that degraded visual acuity impairs performance on the CFMT. This would show that visual acuity contributes to successful face recognition, but it would not establish that visual acuity constitutes a face-identity recognition mechanism. For this reason, I don’t think that the paper make a novel contribution to the field.

Associate Editor
Comments to the Author:
(There are no comments.)

Response to Reviewers
Manuscript: “Anodal Transcranial Direct Current Stimulation (tDCS) at the DLPFC Disrupts Face Recognition Skills” — Oxford Open Neuroscience

We thank the Editor and both reviewers for their careful reading of the manuscript and for their constructive comments, which have substantially improved the paper. Below we reproduce each comment followed by our response. All new or modified text appears in blue in the revised manuscript and Supplementary Material; where helpful, we quote the new text here.

Reviewer 1
Comment 1
The Introduction currently reads as a sequential list of prior studies and their study-specific results, rather than a cohesive, hypothesis-driven narrative. The Introduction should be restructured to condense the current lengthy blocks of text summarizing individual studies into a series of integrated, thematically organized paragraphs… For example, the McLaren perceptual learning model is introduced late in the Discussion, but is not used to help set up the study in the Introduction. Bringing this theoretical framework forward to earlier in the paper would greatly help situate the motivation and potential theoretical significance of this experiment.


Response: We thank the reviewer for this suggestion and have revised the Introduction in two ways. First, we have condensed the previous study-by-study review of the enhancement strand into two integrated, thematically organised paragraphs: the first presents the evidence that anodal tDCS over posterior face-responsive areas can enhance face processing (Barbieri et al., 2016; Brunyé et al., 2017; Saghravanian and Esteky, 2025), and the second presents the moderators of these effects — task demands, montage, and baseline ability (Wu et al., 2021; Kho et al., 2023; Renzi et al., 2015; Willis et al., 2019) — retaining only the study-specific details drawn upon later in the Discussion. The Introduction is thus now organised around the contrast between the two strands (posterior enhancement vs. DLPFC disruption of perceptual learning) rather than around individual studies. Second, we have brought the theoretical framework forward as the reviewer requested: a new paragraph at the end of the Introduction now introduces the McLaren, Kaye and Mackintosh (MKM) model of perceptual learning and derives from it the specific prediction tested in this study. The full mechanistic exposition of the model remains in the Discussion, where it is used to interpret the results.

The new Introduction paragraph reads:
“The theoretical framework guiding this research programme is the McLaren, Kaye and Mackintosh (MKM) model of perceptual learning (McLaren et al., 1989; McLaren and Mackintosh, 2000; McLaren et al., 2012). On this account, experience with a prototype-defined category, such as upright faces, reduces the salience of the features common to its exemplars while leaving each exemplar’s distinctive features relatively salient, thereby improving discrimination between exemplars. The inversion effect arises because this salience-modulation benefit accrues only for the familiar upright orientation, and anodal tDCS at Fp3 is held to disrupt the salience-modulation mechanism itself, selectively impairing upright performance. This framework generates the prediction tested here: if the tDCS procedure acts on the expertise-based learning that supports the individuation of faces, its behavioural signature — disruption of upright but not inverted performance — should extend from image recognition to identity recognition as measured by the CFMT.”

Comment 2
The DLPFC (and the contralateral frontopolar cathode site) are critical domain-general cognitive hubs for numerous processes engaged by this task… While inverted faces are included as a control orientation, baseline recognition for inverted faces is substantially lower (M = 32.5 vs. M = 41.2), meaning this condition does not match the configural working memory load of upright faces. Without a true “positive control” task using a non-face visual category of comparable cognitive difficulty, it is impossible to determine whether these effects are genuinely domain-specific to face recognition per se.

Response: We agree, and we would like to clarify that it was never our intention to claim that these effects are domain-specific to face recognition. On the contrary, our theoretical position — grounded in the finding that the same tDCS procedure modulates the inversion effect for faces and for prototype-defined checkerboards alike — is that the effects reflect perceptual expertise operating through perceptual learning, rather than a face-specific mechanism. In response to this comment (and to Comment 1), the MKM framework is now set out in the Introduction, which we hope makes this standpoint clear from the outset. In addition, the Discussion now states explicitly that the present results do not adjudicate the expertise versus face-specificity debate, and identifies the direct test the reviewer envisages — applying the same procedure to a matched non-face task — as the key future direction, using CFMT-format tests adapted to object recognition such as the Cambridge Car Memory Test (Dennett et al., 2012).

The new Discussion text reads:
“This does not imply that the mechanisms affected are specific to faces: on the perceptual-learning account developed above, comparable effects should obtain for any prototype-defined category sharing a common configuration, and the present results do not directly adjudicate the expertise versus face-specificity debate. What they do show is that a tDCS procedure known to modulate the inversion effect for faces and checkerboards alike extends to a paradigm testing identity recognition rather than stimulus recognition. Future work should apply the same procedure to CFMT-format tests adapted to non-face objects, such as the Cambridge Car Memory Test (Dennett et al., 2012), to address the specificity question directly.”

Comment 3
The study utilizes a bilateral, bipolar tDCS montage with the anode placed over the left DLPFC (Fp3) and the cathode over the contralateral frontopolar/supraorbital cortex (Fp2). Without a flipped montage, an extracephalic return electrode, or another “active control” tDCS condition, it is unclear whether the observed behavioral disruptions are being driven by anodal excitation at the DLPFC, cathodal inhibition at the frontopolar cortex, or both.

Response: This is a very good point, and one that has been addressed empirically across a series of earlier studies in this programme that used active-control tDCS conditions with the same behavioural paradigms. Civile, McLaren et al. (2018) showed that anodal tDCS over the right inferior frontal junction (rIFJ; return/cathode at Fp1) left the FIE as robust as under sham. Civile, McLaren et al. (2021) showed that anodal tDCS over the occipito-parietal area (PO8) with the return/cathode at Fp2 — i.e., holding the return site of the established Fp3–Fp2 montage constant — had no effect. Civile, McCourt et al. (2024) directly compared the anodal Fp3–Fp2 montage against an anodal Fp3–Cz montage, and only the Fp3–Fp2 montage reduced the inversion effects for faces and checkerboards; the Fp3–Cz montage behaved like sham. Together, these studies show that neither moving the anode (with or without holding the Fp2 return constant) nor keeping the anode at Fp3 while moving the return away from Fp2 reproduces the effect: modulation of the inversion effect is obtained only with the anode at Fp3 and the return/cathode at Fp2. We have added a paragraph to the Introduction setting out this evidence in full.

At the same time, we agree with the reviewer that behavioural dissociations of this kind cannot by themselves establish which cortical regions mediate the effect. We have therefore added a limitation paragraph at the end of the Discussion referencing computational modelling of tDCS electric fields, which shows that current density with conventional bipolar montages is spatially diffuse, often not maximal at the nominal target, and highly variable across individuals (Datta et al., 2009; Opitz et al., 2015; Laakso et al., 2015). Consistent with this, we now describe in more detail our stimulation in terms of the electrode montage (anode at Fp3, return at Fp2) rather than an assumed cortical target. This montage-based description has now been implemented throughout the revised manuscript: in the title, which reads “Anodal Transcranial Direct Current Stimulation (tDCS) via the Fp3–Fp2 Montage Disrupts Face Recognition Skills”, and at each point in the Abstract, Introduction, and Discussion where the stimulation was previously described by its assumed cortical target (all changes marked in blue); the DLPFC is retained only at first mention, to indicate the site the montage overlies. We further note that current-flow modelling, individualised stimulation parameters, and combined tDCS–fMRI protocols are the appropriate tools for future work to identify the mediating regions.

The new limitation paragraph reads:
“A final note concerns the anatomical specificity of the tDCS procedure and the question of which cortical areas are affected by the Fp3–Fp2 montage. Computational modelling of the electric fields induced by tDCS has repeatedly shown that the current delivered through conventional bipolar montages is spatially diffuse, and that current density is often not maximal at the nominal cortical target, being shaped by anatomical factors such as skull and cerebrospinal-fluid thickness, gyral depth, and inter-electrode distance (Datta et al., 2009; Opitz et al., 2015). These factors also vary substantially across individuals, producing considerable inter-subject variability in the electric fields induced by the same montage (Laakso et al., 2015). For this reason, we have described our stimulation primarily in terms of the electrode montage adopted (anode at Fp3, return at Fp2) rather than an assumed cortical target. Future work should apply current-flow modelling to this specific montage and, ideally, individualise stimulation parameters based on participants’ anatomy; combined tDCS–fMRI protocols could further help identify the cortical regions mediating the effects reported here.”

Reviewer 2
Comment
Presenting different images is a necessary methodological feature of an identity-recognition task, but it is not sufficient to establish that an experimental manipulation acts specifically upon identity-level representations or identity-recognition mechanisms. Face-identity recognition necessarily depends on earlier image-level processes, including visual analysis, perceptual discrimination, attention to diagnostic facial information, and the encoding of the presented image. If Fp3 stimulation disrupts any of these shared upstream processes… performance will also be impaired on a task requiring recognition across different images… An analogy would be demonstrating that degraded visual acuity impairs performance on the CFMT.

Response: We thank the reviewer for this incisive comment, with which we fully agree: demonstrating that a manipulation disrupts performance on a task requiring recognition across different images does not, by itself, establish that identity-recognition mechanisms are affected. Our response is twofold.
First, we would argue that the selectivity of the effect constrains the upstream account. A manipulation acting on shared upstream processes — the reviewer’s degraded-acuity analogy, or general attentional or encoding disruption — should impair recognition of inverted faces as well as upright ones. Yet anodal tDCS impaired only upright-face recognition, with no effect for inverted faces. Because inversion disrupts configural processing, this selectivity indicates that the stimulation affects, at least in part, a mechanism involved in face recognition — specifically configural processing. This is the logic underlying the use of the inversion manipulation in the original validation of the CFMT (Duchaine and Nakayama, 2006).
Second, the CFMT itself contains a condition that speaks directly to this issue: the novel-images-with-noise phase, designed to force reliance on configural processing, because noise disrupts featural processing while leaving configural processing relatively unaltered (Duchaine and Nakayama, 2006; McKone, Martini and Nakayama, 2001). We have now conducted an additional analysis restricted to this phase, reported in full in the Supplementary Material. Anodal tDCS significantly reduced the FIE in this phase relative to sham (Face Orientation × tDCS: F(1, 62) = 9.05, p = .004, η²ₚ = .13); the inversion effect was robust under sham, t(31) = 4.12, p < .001, d = 0.73, and eliminated under anodal stimulation, t(31) = 0.09, p = .93, and the reduction was driven by impaired upright-face performance, t(62) = 3.66, p < .001, d = 0.92, with no group difference for inverted faces, t(62) = 0.48, p = .62. Thus, even under stimulus conditions specifically designed to isolate configural processing, anodal tDCS disrupted upright-face recognition.

We have added two paragraphs to the Discussion presenting this argument and the new analysis, and acknowledging its limits: these findings do not establish that the affected mechanisms are specific to faces (see also our response to Reviewer 1, Comment 2), and future work should probe the earlier stages of processing directly, for instance using the combined tDCS–EEG methodology (Civile, Waguri et al., 2020, 2024) to examine the effects of stimulation on the inversion effects indexed by the P1, N170, and P250 components.

The first new Discussion paragraph reads:
“A further issue concerns what the present findings can and cannot establish about the distinction between image-level and identity-level processing (Bruce, 1982; Bruce and Young, 1986). We acknowledge that showing that tDCS disrupts performance on a task requiring recognition across different images does not, by itself, demonstrate that identity-recognition mechanisms are affected: identity recognition necessarily depends on earlier image-level processes, including visual analysis, perceptual discrimination, and attention to diagnostic facial information, and a manipulation disrupting any of these shared upstream processes would also impair performance on an identity-recognition task. However, the selectivity of the effect speaks against a purely upstream account. A manipulation acting on general visual or attentional processes should impair recognition of inverted faces as well as upright ones, whereas anodal tDCS impaired only upright-face recognition. Because inversion disrupts configural processing, this selectivity indicates that the stimulation affects, at least in part, a mechanism involved in face recognition — specifically configural processing — which is precisely the logic underlying the use of the inversion manipulation in the original validation of the CFMT (Duchaine and Nakayama, 2006).”

We hope the reviewers find that the revisions have addressed their concerns, and we thank them again for comments that have materially strengthened the manuscript.
