# Supplementary material for: Anodal transcranial direct current stimulation (tDCS) via the Fp3–Fp2 montage disrupts face recognition skills
Source: Oxf Open Neurosci. 2026 Aug 22;5:kvag010. doi: 10.1093/oons/kvag010 (PMC13537463; doi:10.1093/oons/kvag010)
Supplement: Supplementary file 1 [file supplementary_material_kvag010.zip › supplement_REVISED_kvag010.docx]

# Supplementary Material

**Anodal Transcranial Direct Current Stimulation (tDCS) at the DLPFC Disrupts Face Recognition Skills**

## Additional analyses of the individual task phases

To characterise the effect of tDCS across the structure of the task, we analysed recognition performance separately for the three test phases (same images, novel images, and novel images with noise) within each orientation.

### Upright faces

We conducted a 3 × 2 mixed-model ANOVA with Phase (same images, novel images, novel images with noise) as the within-subjects factor and tDCS (anodal, sham) as the between-subjects factor. There was a significant main effect of Phase, *F*(2, 124) = 142.76, *p* < .001, η²ₚ = .70, confirming the intended structure of the task, in which difficulty is highest for the phase involving novel images with noise; noise was added to keep performance away from ceiling (Duchaine and Nakayama, 2006). Mean scores were: same images, *M* = 16.3, *SD* = 2.19; novel images, *M* = 14.9, *SD* = 4.39; novel images with noise, *M* = 9.9, *SD* = 3.13.

Importantly, the Phase × tDCS interaction was not significant, *F*(2, 124) = 1.94, *p* = .15, η²ₚ = .03, indicating that the effect of tDCS did not differ across phases. Critically, there was a significant main effect of tDCS, *F*(1, 62) = 10.09, *p* = .002, η²ₚ = .14, confirming that anodal stimulation reduced overall recognition for upright faces (*M* = 12.8, *SD* = 2.35) relative to sham (*M* = 14.7, *SD* = 2.33).

### Inverted faces

The same analysis was conducted for inverted faces. There was again a significant main effect of Phase, *F*(2, 124) = 47.21, *p* < .001, η²ₚ = .43, reflecting comparable performance in the same-images (*M* = 12.5, *SD* = 2.96) and novel-images (*M* = 11.5, *SD* = 2.54) phases and lower performance in the novel-images-with-noise phase (*M* = 8.4, *SD* = 2.80). The Phase × tDCS interaction was not significant, *F*(2, 124) = 0.55, *p* = .58, η²ₚ = .01, nor was the main effect of tDCS, *F*(1, 62) = 0.07, *p* = .79, η²ₚ < .01, confirming that anodal stimulation did not affect performance for inverted faces.

## Additional analyses of the inversion effect across phases

We computed an inversion-effect index (upright minus inverted) for each phase and conducted a 3 × 2 mixed-model ANOVA with Phase (same images, novel images, novel images with noise) as the within-subjects factor and tDCS (anodal, sham) as the between-subjects factor. There was a significant main effect of Phase, *F*(2, 124) = 6.19, *p* = .003, η²ₚ = .09, indicating a comparable inversion effect in the same-images (*M* = 3.79, *SD* = 3.13) and novel-images (*M* = 3.23, *SD* = 5.26) phases and a reduced inversion effect in the novel-images-with-noise phase (*M* = 1.5, *SD* = 4.20).

Importantly, the Phase × tDCS interaction was not significant, *F*(2, 124) = 2.33, *p* = .10, η²ₚ = .04. Critically, there was a significant main effect of tDCS, *F*(1, 62) = 6.92, *p* = .011, η²ₚ = .10, showing that anodal stimulation reduced the overall inversion effect (*M* = 1.9, *SD* = 2.85) relative to sham (*M* = 3.7, *SD* = 2.62).

**Additional analysis of the novel-images-with-noise phase**

We conducted a 2 × 2 mixed-model ANOVA restricted to the novel-images-with-noise phase, with Face Orientation (upright, inverted) as a within-subjects factor and tDCS (sham, anodal) as a between-subjects factor. There was a main effect of Face Orientation, F(1, 62) = 8.30, p = .005, η²ₚ = .12, with upright faces (M = 9.9, SD = 3.1) recognised better than inverted faces (M = 8.4, SD = 2.8). Critically, the Face Orientation × tDCS interaction was significant, F(1, 62) = 9.05, p = .004, η²ₚ = .13. One-sample t-tests against zero confirmed a robust inversion effect in the sham group (M = 2.9, SD = 3.9), t(31) = 4.12, p < .001, d = 0.73, which was eliminated in the anodal group (M = −0.06, SD = 3.9), t(31) = 0.09, p = .93, d = 0.02. A main effect of tDCS was also found, F(1, 62) = 4.96, p = .030, η²ₚ = .07, indicating lower overall performance in the anodal group (M = 8.6, SD = 2.0) than in the sham group (M = 9.7, SD = 2.1).

An independent-samples t-test showed that performance for upright faces was significantly impaired in the anodal group (M = 8.5, SD = 2.5) relative to the sham group (M = 11.2, SD = 3.2), t(62) = 3.66, p < .001, d = 0.92, whereas performance for inverted faces did not differ between the anodal (M = 8.6, SD = 3.1) and sham (M = 8.3, SD = 2.5) groups, t(62) = 0.48, p = .62, d = 0.12.

## Reference

Duchaine, B., & Nakayama, K. (2006). The Cambridge Face Memory Test: Results for neurologically intact individuals and an investigation of its validity using inverted face stimuli and prosopagnosic participants. *Neuropsychologia, 44*(4), 576–585. https://doi.org/10.1016/j.neuropsychologia.2005.07.001
